# Meal frequency and dietary diversity feeding practices among children 6–23 months of age in Wolaita Sodo town, Southern Ethiopia

**DOI:** 10.1186/s41043-017-0097-x

**Published:** 2017-05-19

**Authors:** Tefera Chane Mekonnen, Shimelash Bitew Workie, Tesfa Mekonen Yimer, Wubalem Fekadu Mersha

**Affiliations:** 1 Department of Public Health, Wollo University, Dessie, Ethiopia; 2 School of Public Health, Wolaita Sodo University, Sodo, Ethiopia; 3 Departments of Psychiatry, School of Medicine, Bahir Dar University, Bahir Dar, Ethiopia

**Keywords:** Dietary diversity, Meal frequency, 6–23 months of children, Southern Ethiopia

## Abstract

**Background:**

Child feeding practices are multidimensional, and they change rapidly within short age intervals. Suboptimal complementary feeding practices contribute to a rapid increase in the prevalence of undernutrition in children in the age of 6–23 months. Information on child feeding practices among urban resident is limited in Ethiopia. The aim was to measure minimum meal frequency and dietary diversity and associated factors among children 6–23 months of age in Wolaita Sodo, Ethiopia.

**Methods:**

A community-based cross-sectional study was carried out to select 623 mothers/caregivers with 6–23 months of children reside in Wolaita Sodo town using systematic sampling from March 02 to 20, 2015. An interviewer-administered questionnaire was used to gather information on socio-demographic, child feeding practices and health-related characteristics. Data were entered to Epi-Data version 3.02 and transported to SPSS version 21 for further analysis. Binary logistic regression was used to see the association between the outcome variables and explanatory variables, and multivariable logistic regression was performed to identify independent predictors of minimum dietary diversity and meal frequency.

**Results:**

The study revealed that the percentage of 6–23 months of children who meet the recommended level of minimum dietary diversity and meal frequency were 27.3 and 68.9%, respectively. Mothers/caregivers who were housewives and government employees feed their children more diversified foods as compared to mothers who were private workers. As compared to children 17–23 months of age, children in the age group of 6–8 and 9–11 months had better probability to meet minimum dietary diversity. Government-employed and illiterate mothers were less likely to feed their children to fulfil the minimum requirement of meal frequency. Children in the age of 9–11 months were also less likely to be fed frequently.

**Conclusions:**

Even though the study showed better progress as compared to the national prevalence of complementary feeding practices, child feeding practices in the study area were inadequate and not achieving WHO infant and young child feeding recommendations. Strengthening the available strategies and creating new intervention measures to improve socioeconomic status, maternal literacy and occupation opportunity for better practices of child feedings are compulsory actions for the government and policymakers.

## Background

Complementary feeding practice is a noteworthy factor that determines the nutritional status of children. The transition period from exclusive breastfeeding to 2 years is a critical window for optimal growth and development of the child. During this period, appropriate, safe, adequately nourished and frequent feeding is essential. Innocently, the food provided to a child might be too high or too low in some nutrients, the diversity of food might be adequate or inadequate and micronutrient content including iron could be lower than required [1].

Suboptimal (inadequate) infant feeding practices are the major reasons for childhood under nutrition in developing countries [2, 3]. Poor nutritional status of children in most developing countries is due to the presence of overwhelming of poverty, low maternal education, high burden of disease and mal-feeding practices [4, 5]. Many survey reports [4–9] consistently indicated that underprivileged child feeding practices are correlated with cultural factors such as selection of low-quality complementary foods, taboos, restrictive traditional beliefs and social factors including caregivers’ poor knowledge on nutrition and lack of knowledge on food diversity in their surroundings. Ultimately, all of these factors lead to low dietary diversity, low feeding frequency and low food and energy intake for children [2, 10].

Several studies have shown that dietary diversity score (DDS) is positively associated with overall dietary quality and micronutrient intake of young children and found to be proxy indicator for household food security and in the long run for childhood stunting [6, 11]. A higher DDS has also been associated with better nutritional status of children in developing countries [12, 13].

As per the recommendation of World Health Organization/Pan American Health Organization (WHO/PAHO) 2003, breastfed children 6–23 months should receive animal-source foods and vitamin A-rich fruits and vegetables daily. Therefore, four food groups (grain- or tuber-based staple, animal-source food, vitamin A-rich fruit or vegetable) are considered the minimum acceptable number of food groups for breastfed infants. Non-breastfed children should be fed meals four or five times per day, with one to two snacks as desired. Meal frequency is considered a proxy for energy intake from foods other than breast milk. Therefore, for non-breastfed children feeding frequency indicators include both milk feeds and solid or semi-solid feeds [14, 15].

Updated knowledge of feeding practices will assist the national nutrition programme to monitor the changes in the feeding practices and design interventions to increase the recommended feeding practices and thereby contribute in reducing undernutrition in the country. In general speaking, Ethiopia is known to have low minimum dietary diversity (MDD) as compared to the rest of the world. Some surveys including national demographic health survey of 2011 conducted in Ethiopia revealed that MDD is below 5%. Five years back, the magnitude of MDD, minimum meal frequency (MMF) and minimum acceptable diet in Southern Ethiopia were 3.8, 49.8 and 3.1%, respectively [16]. Even though there are limited studies done in Ethiopia which were primarily focusing on measuring complementary feeding levels in the rural communities, the current study was unique because it was designed to be conducted in urban area of Ethiopia. The existing surveys are found mostly in Northern and Western parts of Ethiopia [17]. No survey was investigated on dietary diversity and meal frequency in Southern Ethiopia particularly in the urban areas. There should be an urgent measure to identify reasons why complementary feeding indicators are still low. This study was aimed to measure the proportions of minimum dietary diversity and minimum meal frequency and to investigate factors associated with them among young children aged 6–23 months.

## Methods

### Study area, study design and participants

Community-based cross-sectional study design was carried out in Wolaita Sodo town on March 02–20, 2015. The town is found in Wolaita zone, Southern Ethiopia, and 315 km far away from Addis Ababa. The town is administratively structured by 11 kebeles and has a total population of 110,660, of which 54,275 are males and 56,385 are females. Out of all female population, 25,784 of them are women in the reproductive age group. About 14% of the total population are children 6–59 months of age.

Mothers or caregivers of children 6–23 months of age who reside in Wolaita Sodo town were the source population, whereas mothers/caregivers of children 6–23 months that were drawn from the selected kebeles were considered as the study population. Mothers/caregivers of children 6–23 months of age who have been residents of the town and have ever breastfed in the selected kebeles were included in the study. However, mothers or caregivers who are seriously ill, mental problem or unable to communicate were excluded.

### Sample size and sampling procedure

The sample size was determined using single proportion population formula by assuming the proportion of introduction of complementary foods as 42.9% in western Ethiopia [17], 1.5 as design effect, 5% as level of significance and 5% as degree of precision and which was 566. Adding 10% as non-response rate, 623 mothers/caregivers with 6–23 months of children were included in the study. To ensure the adequacy of sample size, Epi-info was used to calculate sample size for factors associated with minimum dietary diversity and minimum meal frequency. Then, the maximum sample size was taken.

Two stage sampling was used to select the participants. Seven administrative units were randomly drawn from 11 administrative units. A census was conducted in these selected kebeles to identify the study participants. The sample size was allocated to the population size proportionately, and sampling interval was calculated. Finally, 623 participants were selected using a systematic sampling method after randomly identified the first household and proceed to the second participant based on the interval.

### Data collection and measurements

Data were collected using interviewer-administered validated questionnaire in a face-to-face manner from mothers/caregivers of children 6–23 months of age. The questionnaire consists of three parts: socio-demographic characteristics of households, maternal and child health related features and child feeding practices. Twenty-four-hour recall method and food frequency questionnaire were used to assess dietary diversity and meal frequency. Ten first degree in nursing holders as data collector and three second degree holders who had previous experiences as supervisor were involved in the survey.

Structured questionnaire partly adopted from WHO assessment tool for infant and young child feeding (IYCF) practices were used and translated into local language by fluent speakers and back translated to English to validate the consistency. Training was given for data collectors and supervisors on how to interview and maintaining the quality of data. Pre-test was done on 5% of the participants out of the selected areas. Then, the questionnaire was rechecked for its precision and consistency, and necessary modifications were incorporated before commencing the actual data collection. The supervisors and the investigatory were regularly monitored and checked the completeness of the data in daily bases.

#### Minimum dietary diversity

Proportion of children 6–23 months of age who receive foods from four or more food groups during the previous day. The seven food groups used for tabulation of this indicator were as follows: cereals, roots and tubers; legumes and nuts; dairy products (milk, yoghurt and cheese); flesh foods (meat, fish, poultry and liver/organ meats); eggs; vitamin A-rich fruits and vegetables and other fruits and vegetables [14].

#### Minimum meal frequency

Proportion of breastfed and non-breastfed children 6–23 months of age, who receive solid, semi-solid or soft foods (but also including milk feeds for non-breastfed children) the minimum number of times or more. Breastfed infants of age 6–8 and 9–23 months should obtain a minimum of two or three meals with one to two snacks and three or four meals with one to two snacks per day, respectively. But non-breastfed infants of age 6–23 months should receive milk products at least twice a day [8].

### Statistical analysis

Data were entered to Epi-Data 3.02 and exported, cleaned and analysed by SPSS version 21. Missed data were explored, and normality for continuous variables was checked. Dietary diversity score (DDS) was computed out of seven from seven food groups. Reliability of the tool was done, and Cronbach’s Alpha value was 0.76. Household economic status was measured by constructing a wealth index through principal component analysis. The indicator variables used for wealth index construction that fulfil the requirement of factor analysis were telephone, table, chair, refrigerator and electric mitad. Varimax rotation was used. The communality of each variable was greater than 0.53; Kaiser-Meyer-Olkin measure of sampling adequacy was 0.58. The cumulative proportion of variance criteria was met with two components which was 66.80%. Split sample validation was done, and none of communality’s of the variable in each split was below 0.5 and finally categorized into poor, medium and rich.

The data were presented in tables and figures by computing the percentages of minimum dietary diversity, meal frequency and acceptable diet. Binary logistic regression was done for the two outcome variables of MDD (1 = met 4 and above food groups, 0 = met less than four food groups) and MMF (1 = met the minimum requirement for age groups, 0 = not fulfil the minimum requirement). The strength of associations and statistical significances between explanatory variables and outcome variables were expressed using OR and 95% of confidence interval, respectively.

All variables in the binary logistic regression with *p* value <0.1 were moved to multivariable logistic regression and done using backward likelihood ratio to control the possible confounders and to identify predictors of the outcome variables. At this level, model fitness was checked which was Hosmer-Lemonshow as 0.67 and no multicollinearity. The variable was considered to be predictive for each outcome variable at *p* value of less than 0.05.

## Results

### Characteristics of participants

There were 611 mothers/caregivers with children 6–23 months, which constitute 98.1% of response rate. From all participants, 605 (99%) of them were biological mothers. The mean age of mothers/caregivers was 26.7 years with ± 4.8 years of standard deviation, and the median age was also 26 years. Two third (65%) of the respondents’ occupational status were found to be as house wives, and about a quarter of the participants (26%) were accomplished grade 10 and colleges but 11.5% of mothers/caregivers had no education (Table 1).

**Table 1:** Socio-demographic, economic and other characteristics of the participants reside in Wolaita Sodo town, Ethiopia, in 2015

Variables (*n* = 611)	Frequency (%)
Household head	
Male	570(93.3)
Female	41(6.7)
Maternal and caregivers age (years)	
15–19	18(3.0)
20–24	166(27.2)
25–29	263(43.0)
30–34	109(17.8)
≥35	55(9.0)
Maternal relation with child	
Mother	605(99.0)
Caregiver	6(1.0)
Marital status	
Single	3(0.5)
Married	577(94.4)
Divorced	22(3.6)
Widowed	9(1.5)
Household head occupation	
House wife	30(4.9)
Government employee	184(30.1)
Daily worker	144(23.6)
Private worker	253(41.4)
Maternal occupation	
Housewife	397(65.0)
Private	91(14.9)
Government	123(20.1)
Educational status of woman	
Illiterate	70(11.5)
Grade 1–8	218(35.7)
Grade 9–10	164(26.8)
Above 10	159(26.0)
Wealth index	
Poor	433(70.9)
Middle (medium)	72(11.8)
Rich	106(17.3)
Sex of child	
Male	351(57.4)
Female	260(42.6)
Age of child (in months)	
6–8	112(18.3)
9–11	118(19.3)
12–17	249(40.8)
18–23	132(21.6)
ANC follow-up	
Yes	595(97.4)
No	16(2.6)
PNC follow-up	
Yes	469(76.8)
No	142(23.2)
Place of delivery	
Home without TBA	25(4.1)
Home with TBA	12(2.0)
Government health facility	523(85.6)
Private health facility	51(8.3)

### Complementary feeding practices

**Fig. 1:**
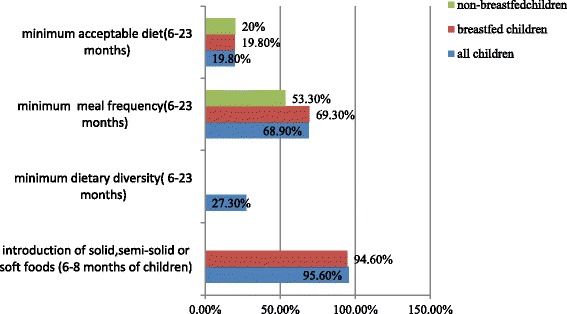
Indicators of complementary feeding practice of children 6–23 months of age in Wolaita Sodo town, Ethiopia, in 2015

The overall children who met the requirement of minimum dietary diversity were 27.3% ranged from 23.7–30.8% at 95% CI and minimum meal frequency for both breastfed and non-breastfed children were 68.9% which lied within 65.2–72.6% at 95% CI. Moreover, those who met the requirement of a minimum acceptable diet were 21.1% (Fig. 1).

There was a clear observation that dietary diversity decreases as age increases but as age increases meeting the requirement of meal frequency increases. Significant numbers of 6–8 months of children (50.9%) were met the requirement of a minimum dietary diversity as compared to children 18–23 months of age (22.3%) (Table 2).

**Table 2:** Percentage distribution of minimum dietary diversity, meal frequency and acceptable diet disaggregated by age of children 6–23 months in Wolaita Sodo town, 2015

Child age category	Meet minimum dietary diversity (*n* = 611)	Meet minimum meal frequency (*n* = 611)	Meet minimum acceptable diet (*n* = 611)
6–8 months	50.9%	65.2%	33.9%
9–11 months	28.8%	62.7%	19.5%
12–17 months	18.1%	69.3%	17.2%
18–23 months	22.3%	75.3%	18.7%
Over all practice with 95% CI	27.3% (23.7–30.8%)	68.9% (65.2–72.6%)	21.1% (17.8–24.3%)

**Fig. 2:**
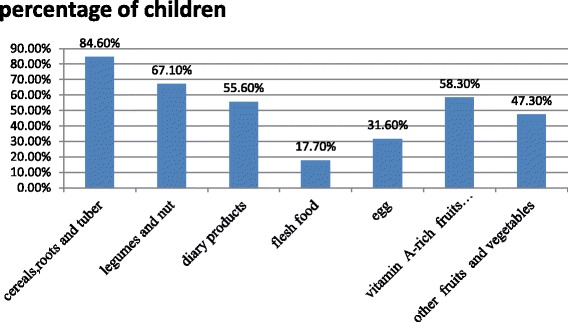
The proportion of 6–23 months of children who consumed a variety of food groups in Wolaita Sodo town, Ethiopia, in 2015

The majority of children (84.6%) consumed grains, roots and tubers and followed by legumes and nuts (Fig. 2) in the past 24 h prior to the data collection. However, smaller proportions of children were consumed fish (9.7%) and iron-rich food (liver) 1.1%.

### Factors associated with minimum dietary diversity and meal frequency

Among the variables reached to the final model, household head, occupation and child age were statistically associated with minimum dietary diversity. Children from households headed by housewives were 2.3 times more likely to be fed four and above food items or groups per day as compared to children from households headed by private workers [adjusted odds ratio (AOR) = 2.3; 95% CI (1.01–5.4)]. In addition, the odds of meeting minimum dietary diversity among children from families headed by government workers were almost four times higher than the odds of minimum dietary diversity among children from families headed by private workers [AOR = 3.7; 95% CI (2.3–5.9)]. The odds’ of minimum dietary diversity in children 6–8 months of age were nearly five times higher than the odds’ of minimum dietary diversity among older children [AOR = 5.2; 95% CI (2.9–9.1)].

Likewise, among the variables reached the final step, household head occupation, maternal/caregiver educational level, child sex and age were identified as predictors of minimum meal frequency. The chance of children came from households lead by government workers, who met the requirement of minimum meal frequency, were reduced by 40% [AOR = 0.6; 95% CI (0.4–0.9)]. The probability of meeting the requirement of minimum meal frequency was reduced by 50% if the child fed by illiterate mothers/caregivers as compared to mothers or caregivers who attain grade 10 and above [AOR = 0.5; 95% CI (0.2–0.9)]. Girls were nearly two times more likely to be fed frequently as compared to boys [AOR = 1.5; 95% CI (1.02–2.1)] (Table 3).

**Table 3:** Backward multivariable logistic regression used to identify predictors of complementary feeding practices (MDD and MMF) in children 6–23 months at Wolaita Sodo town, Ethiopia, in 2015 with 95% of confidence interval

Variables	Meet MDD (*n* = 611)			Meet MMF (*n* = 611)		
	*n*	COR	AOR	*n*	COR	AOR
Household head						
Male	154	0.8(0.4–1.6)	^a^	391	0.8(0.4–1.6)	^a^
Female	13	1		30	1	
Maternal age						
≤24 years	54	1.5(0.8–2.7)	^a^	132	0.8(0.5–1.5)	^a^
25–30 years	94	1.4(0.7–2.4)		223	0.6(0.4–1.1)	
≥31 years	19	1		66	1	
Marital status						
Single	13	1.7(0.8–3.5)	^a^	26	1.5(0.7–3.4)	^a^
Married	154	1		395	1	
House hold head occupation						
House wife	12	*2.4*(*1.1–5.3*)	*2.3*(*1.01–5.4*)	25	2.1(0.8–5.8)	2.0(0.7–5.6)
Government employee	31	*3.3*(*2.1–5.2*)	*3.7*(*2.3–5.9*)	112	*0.7*(*0.4–0.9*)	*0.6*(*0.4–0.9*)
Daily worker	69	0.7(0.4–1.2)	0.7(0.4–1.2)	107	1.2(0.8–1.9)	1.3(0.8–2.2)
Private worker	55	*1*	1	177	1	1
Maternal occupation						
House wife	120	1.6(0.9–2.6)		276	1.1(0.7–1.7)	
Government employee	21	1.1(0.6–2.1)	^a^	62	1.0(0.6–1.8)	^a^
Private worker	26	1		83	1	
Maternal education						
Illiterate	32	0.9(0.6–1.4)	1.5(0.6–2.7)	45	0.8(0.4–1.4)	*0.5*(*0.2*–*0.9*)
Up to grade 10	94	*2.3*(*1.3*–*4.4*)	0.7(0.4–1.1)	265	0.9(0.6–1.5)	0.8(0.5–1.2)
Above grade 10	41	1	1	111	1	1
Wealth index						
Poor	121	1.3(0.7–2.0)		308	1.5(0.9–2.3)	
Middle	21	1.3(0.6–2.6)	^a^	47	1.1(0.6–2.1)	^a^
Rich	25	1		66	1	
Child sex						
Male	94	1	^a^	229	1	1
Female	73	1.1(0.7–1.5)		192	*1.5*(*1.1*–*2.1*)	*1.5*(*1.02*–*2.1*)
Child age (months)						
6–8	57	*3.6*(*2.1*–*6.1*)	*5.2*(*2.9*–*9.1*)	73	0.6(0.4–1.0)	0.6(0.3–1.02)
9–11	34	1.4(0.8–2.4)	*1.8*(*1.01*–*3.3*)	74	*0.6*(*0.3*–*0.9*)	*0.5*(*0.3*–*0.9*)
12–17	39	0.7(0.5–1.3)	0.9(0.6–1.7)	149	0.7(0.5–1.2)	0.7(0.5–1.2)
18–23	37	1	1	125	1	1
ANC follow-up						
Yes	160	0.5(0.2–1.3)	^a^	408	0.5(0.1–1.8)	^a^
No	7	1		13	1	
Place of delivery						
Home delivery	15	1	^a^	26	1	^a^
Health facilities	152	0.5(0.3–1.0)		395	0.9(0.4–1.9)	

The italic values show statistically significant variables

*COR* crude odds ratio, *AOR* adjusted odds ratio, *MDD* minimum dietary diversity, *MMF* minimum meal frequency

^a^Variables in the model not reached final step

## Discussion

The survey publicized that the proportions of minimum dietary diversity and minimum meal frequency were 27.3 and 68.9% in all 6–23 months of children, respectively. In this study, minimum dietary diversity and meal frequency were higher than findings from northwest part of Ethiopia (with MDD of 12.6% and MMF of 50.4%) and also higher than results analysed from EDHS 2011 which showed that 10.8 and 44.7% of children meet MMD and MMF, correspondingly [16, 17]. The proportion of 6 to 23 months of children who met the recommended level of meal frequency in the study were nearly two times more than report from demographic health survey data analysis from Tanzania and Uganda but with nearly similar report with dietary diversity [7]. And these core indicators were also higher than findings from WHO 2010 reports in Eretria (19 and 44%), Guinea (18 and 30%), India (12 and 44%), Niger (5 and 42%) and Mali (16 and 25%) but less than from Kenya (45 and 58%), Zambia (37 and 55%), Indonesia (65 and 67%) and Morocco (66 and 62%) for MDD and MMF, respectively [15]. The reason for the high percentage of child feeding practice in the study area may be due to the variation in time of data collection, and nutrition education now a day may play a vital role in increasing the awareness of community for better feeding practices in urban settings. Likewise, the result of the study was almost consistent with survey conducted in Nepal among 6 to 23 months of children which revealed that 76.6 and 30.4% of them obtained MMF and MDD, respectively [8].

In this study among variables moved to the final model, occupation of household head, child age, maternal education and child sex were found to be statistically associated with complementary feeding practices. Household head occupation being government-employed and house wife, younger children were more likely met the recommended dietary diversity. The finding was supported by report from Nepal [8]. Probably, housewives spent their entire times with their children and care them in a better manner. Government-employed mothers/caregivers are also more educated and attentive and have plan to prepare diversified diets.

Children from household headed by government-employed mothers and illiterate mothers or caregivers were less likely to meet the minimum requirement of meal frequency. Girls were more frequently take meals as compared to boys. Children in the age of 9–11 months were less likely to meet recommended meal frequency as compared to older children [7, 17]. But a report from Uganda [9], maternal education has no association with child feeding practices. Educated mothers or caregivers are easier to be familiarized with new information and knowledge, and they know the importance of child feeding practices as compared to illiterate one who are stagnant and needs longer time to bring behavioural change. This study indicated that government-employed mothers/caregivers fed their children more diversified diet but less frequent. This is due to employed mothers/caregivers stayed at work place separated from their children for long time.

Eventually, this survey had its own strength and limitation. Tremendous efforts were made to assure the quality of the study starting from the period of predata collection to write-up of the report. Reliability of the tool was checked, and appropriate statistical test was performed for different model assumptions. Nevertheless, the study had unforgettable limitations. The study used only 24-h recall method which tells us only one time phenomenon but did not demonstrate dietary habit of the participants and affected by variation of days. Moreover, the study fails to address measuring of mean dietary adequacy, the mean density of nutrient and finally did not show the relationship of these feeding practices to nutritional status of children.

## Conclusions

The study revealed that the percentage of children who meet the recommended level of minimum dietary diversity, meal frequency and acceptable diet were 27.3, 68.9 and 21.1%, respectively. These are good achievements as compared to the national figures but surprisingly insignificant as weighted against countries who are found in a better standard of living condition. Particularly minimum dietary diversity and acceptable diet in the study site were inadequate.

Child feeding practices in the study area were significantly influenced by household occupational status, maternal education, child sex and age, and socioeconomic status of the household. And thus, they were identified as independent factors that made inadequate complementary feeding practices. In contrast to this, there were some important factors that are not statistically significant with child feeding practices.

Despite of the observed better achievement as compared the national proportion of complementary feeding indicators, much is expected to reach the target of the health sector development programme of the country as well as the WHO recommendation level. Emphasis should be undertaken to increase maternal literacy and employment opportunity to have better economic status because they are the proxy and decisive stakeholders for superior accomplishment of reaching the target. It is better to conduct further research that focuses on the relationship of these infants and young child feeding practices with child nutritional status.

## Abbreviations

AOR: Adjusted odds ratio
COR: Crude odds ratio
DDS: Dietary diversity score
EDHS: Ethiopia Demographic Health Survey
IYCF: Infant and young child feeding
MDD: Minimum dietary diversity
MMF: Minimum meal frequency
